# The DARO-flare trial: evaluating the impact of darolutamide on prostate-specific membrane antigen (PSMA) flare and its implications for imaging and staging of hormone-sensitive prostate cancer – study protocol

**DOI:** 10.1136/bmjopen-2025-115888

**Published:** 2026-06-18

**Authors:** Suzanne van der Gaag, André Vis, Imke Bartelink, Harry Hendrikse, Daniela E Oprea-Lager

**Affiliations:** 1 Department of Radiology & Nuclear Medicine, Amsterdam UMC Location VUmc, Amsterdam, The Netherlands; 2 Imaging and Biomarkers, Cancer Centre Amsterdam, Amsterdam, The Netherlands; 3 Department of Urology, Amsterdam UMC Location VUmc, Amsterdam, The Netherlands; 4 Department of Clinical Pharmacology and Pharmacy, Amsterdam UMC Location VUmc, Amsterdam, The Netherlands; 5 Department of Medical Imaging, Radboud University Medical Center, Nijmegen, The Netherlands

**Keywords:** Prostate disease, Urological tumours, NUCLEAR MEDICINE

## Abstract

**Introduction:**

Prostate cancer (PCa) represents a major global health concern due to biological heterogeneity and the complexity of its underlying molecular pathways, which contribute to substantial morbidity and mortality. Ongoing research continues to elucidate distinct molecular mechanisms that may inform diagnostic and therapeutic strategies. Among these, the prostate-specific membrane antigen (PSMA) has emerged as a pivotal biomarker and therapeutic target in PCa. PSMA is indirectly modulated by androgen signalling through the androgen receptor (AR) pathway and has been observed to undergo transient upregulation following treatment with AR targeting agents, such as darolutamide, particularly in patients with castration-resistant PCa. This transient increase in PSMA expression—termed the ‘PSMA flare phenomenon’—may enhance the sensitivity and accuracy of PSMA-based molecular imaging and disease staging.

**Methods and analysis:**

The DARO-flare trials are a prospective, single-centre, interventional, phase I–II proof-of-concept study evaluating whether short-term administration of darolutamide induces a PSMA-flare in patients with hormone-sensitive PCa. The cohort being investigated comprises patients with oligometastatic recurrence following prior therapy with curative intent.

Within the cohort, participants are randomised (1:1) to receive darolutamide 600 mg two times a day for either 7 or 14 days. All participants undergo baseline PSMA positron emission tomography (PET)/CT imaging as standard-of-care and two follow-up scans on days 7 and 14.

The primary endpoint was the change in the number of PSMA-expressing PCa lesions and/or PSMA expression per lesion maximum standardised uptake value on repeated PSMA PET/CT, indicative of a darolutamide flare response. Secondary endpoints included the impact on clinical management decisions, defined as a treatment change following short-course darolutamide, tolerability and toxicity as assessed by adverse event reporting, and patient-reported outcomes related to quality of life.

**Ethics and dissemination:**

The study protocol has been approved by the Medical Ethics Committee (METC) Amsterdam UMC and adheres to the Declaration of Helsinki. Written informed consent is obtained from all participants. Study findings will be disseminated through peer-reviewed publications and conference presentations.

**Trial registration number:**

2025-520482-52-03 (https://euclinicaltrials.eu/ctis-public/view/2025-520482-52-03?lang=en, protocol nr 6.0).

**STRENGTHS AND LIMITATIONS OF THIS STUDY**

- This is a prospective single-centre phase I–II interventional study using a randomised design between two exposure durations.
- Serial prostate-specific membrane antigen (PSMA) positron emission tomography/CT imaging at three predefined timepoints enables evaluation of temporal kinetics of PSMA upregulation.
- Imaging interpretation is based on standardised PSMA PET/CT Analysis and Reporting Consensus (SPARC) criteria and dual-reader assessment.
- The study is limited by its small sample size and exploratory, non-confirmatory design.
- The single-centre design may limit the generalisability of the findings.

## Introduction

 Prostate cancer (PCa) remains a major global health concern, characterised by its biological heterogeneous nature and complex androgen-dependent signalling pathways. The prostate-specific membrane antigen (PSMA), a transmembrane glycoprotein highly expressed in malignant prostate tissue, has emerged as a promising theranostic target, serving both diagnostic and therapeutic applications.

PSMA is indirectly regulated through the androgen receptor (AR) pathway. Under normal physiological conditions, androgens such as testosterone are converted to dihydrotestosterone (DHT), which forms a DHT-AR complex that suppresses the folate hydrolase 1 (*FOLH1)* gene, thereby reducing PSMA protein synthesis. Inhibition of this signalling by androgen receptor targeting agents (ARTAs), including darolutamide, enzalutamide and apalutamide, reverses this suppression mechanism and results in upregulation of PSMA expression, a phenomenon referred to as the ‘flare phenomenon’.^1^

The PSMA-flare phenomenon, characterised by enhanced PSMA protein expression, holds promise for improving PCa imaging and therefore staging. Preclinical studies using human PCa cell lines, such as LNCaP and C4-2, have demonstrated concentration-dependent PSMA upregulation on exposure to ARTAs.^1^,^8^ Furthermore, clinical evidence from human case reports and prospective studies corroborates these findings, with short-term ARTA treatment leading to increased maximum standardised uptake value (SUV_max_) on PSMA-positron emission tomography (PET/CT) imaging.^9^,^17^ The optimum timeframe for ARTAs to exhibit the flare phenomenon appears to range between 9 and 14 days.^13^,^16^ Interestingly, the flare phenomenon is confined to PSMA-expressing target organs and is not observed in organs-at-risk, which are responsible for potential side effects.^9^,^16^

Despite structural differences among ARTAs, their pharmacokinetic behaviour and AR antagonism are comparable, and it is reasonable to anticipate no alteration in their mechanism of action. Preclinical data suggest a consistent flare phenomenon across various ARTAs, with darolutamide demonstrating similar upregulation compared with enzalutamide.^8^

This study aims to investigate the effect of ARTAs, specifically darolutamide, on the upregulation of PSMA (the PSMA flare) in patients at various stages of hormone-sensitive PCa. The potential impact of this transient PSMA flare on molecular imaging modalities may contribute to more accurate disease detection and staging.

Enhanced staging precision could, in turn, inform treatment selection, optimise therapeutic planning and ultimately improve patient outcomes and quality of life.^18^

## Methods and analysis

### Study setting

This study represents a prospective, single-centre, interventional, phase I–II proof-of-concept study (2025–520482-52-03) conducted at Amsterdam UMC, location VUmc. The study is planned to start in January 2026 and is to be completed in December 2026. The following cohort is studied:

- Patients with oligometastatic recurrence (≤5 lesions) after curative-intent therapy.

Within the cohort, patients are randomised (1:1) to receive darolutamide 600 mg two times a day for 7 or 14 days (open-label). PSMA PET/CT is repeated on days 7 and 14 for all patients.

### Population and eligibility criteria

The study population comprises patients diagnosed with PCa, with oligometastatic recurrence (≤5 lesions) detected on re-staging PSMA PET/CT following curative-intent treatments like robot-assisted radical prostatectomy (RARP) or external beam radiation therapy (EBRT). Baseline staging before definitive treatment was performed according to standard clinical practice. Histopathological characteristics, including histological subtype, are recorded in the electronic patient record.

Inclusion criteria require participants to be adult men (>18 years), with histologically confirmed PCa, an Eastern Cooperative Oncology Group performance status of 0–1, and a life expectancy exceeding 12 months. Patients must not have received prior hormonal therapy, must be able to understand the patient information form, and must have provided written informed consent. In addition, eligible patients must have previously undergone RARP or EBRT, and have a PSMA PET/CT demonstrating 1–5 metastatic lesions in bones and/or lymph nodes, irrespective of prostate-specific antigen (PSA) level, grade or disease stage.

Exclusion criteria include non-adenocarcinoma histology, prior PSMA-based radioligand therapy, prior use of ARTAs within the preceding 3 months, presence of visceral or central nervous system metastases, concurrent cytotoxic chemotherapy, immunotherapy, radioligand therapy or investigational therapy, and other specified medical conditions that could impact trial participation (see Concomitant medication section). Additional exclusions include known hypersensitivity to darolutamide or related compounds, prior hip replacement surgery, Sjogren’s syndrome, the presence of second active malignancies or unwillingness to use medically acceptable forms of barrier contraception.

### Intervention

Darolutamide (Nubeqa) is administered orally at 600 mg two times a day with food for either 7 or 14 days, based on treatment arm. Treatment is followed by a 21-day follow-up period, during which two follow-up PSMA PET/CT scans with (^18^F)F-Piflufolastat (Pylclari) will be performed in an outpatient setting.

### Concomitant medication

Any medication that is considered necessary and will not interfere with the study medication or its outcomes may be given at the investigator’s discretion. Other (anti)androgenic treatment may influence the flare phenomenon and are not allowed during the study period. Darolutamide is a substrate for cytochrome P450 (CYP) 3A4, P-glycoprotein (P-gp), breast cancer resistance protein (BCRP) and uridine diphosphate-glucuronosyltransferase (UGT) 1A9. The drug inhibits BCRP, organic anion transporting polypeptide (OATP) 1B1 and OATP1B3 and is a small inducer of CYP3A4. The use of strong CYP3A4 inhibitors may increase the systemic exposure to darolutamide and are therefore prohibited. Likewise, strong CYP3A4 inducers may decrease the exposure of darolutamide. All other concomitant treatments will be registered in the eCRF.

### Criteria for defining the flare phenomenon

The flare phenomenon is defined as follows (table 1): a PSMA flare is identified by the appearance of at least one new PSMA-expressing lesion as compared with baseline imaging, using a liver uptake-specific threshold, according to the Standardised PSMA PET/CT Analysis and Reporting Consensus (SPARC) reporting consensus.^19^ These new lesion(s) may represent either local recurrent disease or new metastatic deposits. Additionally, a PSMA-flare phenomenon can be defined as a clinically significant increase in SUV_max_ and mean standardised uptake value (SUV_mean_) of pre-existing lesions, defined as ≥20%, relative to baseline PSMA PET/CT. Finally, progression from oligometastatic to poly-metastatic disease on repeat PSMA PET/CT. Poly-metastatic disease is defined as the presence of more than five lymph node or bone metastases on follow-up imaging, compared with ≤5 lesions on baseline PSMA PET/CT.

**Table 1:** Overview of PSMA flare criteria in the patient cohort

Baseline PSMA PET/CT	PSMA flare criteria
Oligometastatic (≤5 lesions)	≥1 new PSMA-positive lesion with SUV_max_ and SUV_mean_ >liver uptake-specific threshold ≥20% increase in SUV_max_ and SUV_mean_ of existing lesions New lesions may be local recurrence or metastases Conversion from oligometastatic (≤5 lesions) to poly-metastatic (>5 lesions) disease

PET, positron emission tomography; PSMA, prostate-specific membrane antigen; SUVmax, maximum standardised uptake value; SUVmean, mean standardised uptake value.

### Objectives

The primary endpoint was to prospectively investigate whether the ‘darolutamide flare phenomenon’, observed after the administration of the second line ARTA darolutamide in patients with PCa, results in (1) a higher number of PSMA expressing PCa deposits, either defined as local residual disease or metastatic disease, or (2) in a higher expression of PSMA in individual lesions, determined by SUV_max_, on PSMA PET/CT.

The secondary objectives were (1) to assess the absolute number and the relative number of patients in whom a treatment change is observed by renewed imaging by PSMA PET/CT after a short (7 or 14 days) course of administration with darolutamide, and (2) to assess the patient-reported toxicity and side effects of a short course of administration with darolutamide.

### Sample size

**Figure 1:**
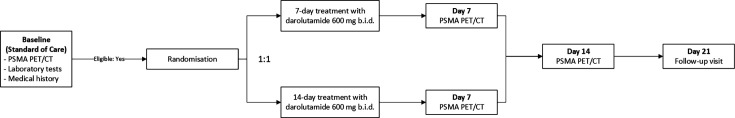
Flowchart of the study design. b.i.d., two times a day; PET, positron emission tomography; PSMA, prostate-specific membrane antigen.

A total of 16 patients will be enrolled, with eight participants per treatment arm (figure 1). The decision to include eight patients per study group is based on a confidence level of 95% and an estimated event probability of 32%, derived from prior studies reporting an average PSMA upregulation of approximately 40% (95% CI 1% to 110%). This sample size is pragmatic and aligns with typical early proof-of-concept oncology studies, focusing on biological feasibility rather than hypothesis testing. Data generated from this study will inform statistical powering of future randomised trials.

### Data collection methods

#### PSMA PET/CT imaging

Patients will be staged with (^18^F)F-piflufolastat (Pylclari) which will be synthesised via direct radiofluorination under Good Manufacturing Practice conditions at an on-site cyclotron facility. Patients will receive 300±10% MBq of (^18^F)F-piflufolastat. PSMA PET/CT imaging will be performed according to local protocol on a European Association of Nuclear Medicine (EANM) Research Ltd. (EARL)-accredited total body PET system and in line with the EANM guidelines on PCa imaging.^19 20^ Protocol started with a low-dose CT scan (50 mAs, 120 kV), followed by a PET scan 120 min after injection. PET images will be acquired from mid-thigh to skull-base. To mitigate radiation exposure, all scans are performed using low-dose CT protocols and optimised PET acquisition according to As Low As Reasonably Achievable (ALARA) principles, and the cumulative exposure of three scans was reviewed and approved by the medical ethics committee as acceptable for this short-term study.

For each scan, PET data were reconstructed using the default blob ordered-subsets time-of-flight reconstruction algorithm, providing images with a matrix of 144×144×45 voxels and with an isotropic voxel size of 4 mm. All PET-images will be corrected for scatter, decay and random coincidences. The PSMA PET/CT scans will be interpreted by both a nuclear physician and an independent reviewer in line with the SPARC guidelines.^21^ Lesion number, location, SUV_max_, SUV_mean_, PSMA expression in the prostate, prostate bed or metastases (lymph node and/or bone metastases), and lesion reporting adhering to molecular imaging TNM classification as per SPARC criteria will be assessed.^21^ Visual and quantitative assessments will be provided, detailing suspected cancer sites, location, size and number. Additionally, findings will be classified on a validated 5-point scale to indicate the likelihood of PCa presence.

#### Blood sample collection

Blood samples will be drawn at the outpatient department on day 0, as standard-of-care, including evaluations of haemoglobin/haematocrit, creatinine, aspartate aminotransferase/alanine aminotransferase, alkaline phosphatase, PSA and testosterone levels.

#### Adverse event monitoring

Adverse events (AEs) and serious AEs (SAEs) are recorded from informed consent until end-of-study and graded per National Cancer Institute Common Terminology Criteria for Adverse Events (CTCAE V.5.0). Expected short-term side effects of darolutamide include mild fatigue and hot flushes (1%–10%).

#### Quality of life assessment

Quality of life will be assessed using quality of life questionnaires (patient-reported outcome measures) from the European Organisation for Research and Treatment of Cancer, at baseline and at end of follow-up.

#### Data management and confidentiality

A data management plan, adhering to the findable, accessible, interoperable and reusable (FAIR) principles, will be implemented following the guidelines of the Amsterdam University Medical Center Clinical Research Bureau (CRB). Restricted access to data will be enforced, and an electronic log will be maintained to track patients meeting eligibility criteria, those invited to participate, recruited patients and those who withdraw from the study, with reasons for non-recruitment documented. Reasons for withdrawal and failure to follow-up will also be documented during the study period. Data will be electronically stored in case report forms (CRFs) software equipped with audit trail functionality, subject to audit by the institutional CRB. The electronic CRF approved by Good Clinical Practice (GCP) standard will be sourced from Castor EDC (Castor EDC, Amsterdam, The Netherlands).

Each participant will be assigned a study code in the electronic CRF software based on application order for de-identification purposes. Only de-identified information will be stored, and participants will be identifiable solely by their unique study number, stored separately. Data will be retained on servers for 25 years in accordance with national guidelines, with the principal investigator granted access to the final trial dataset. Data generated in this study will be made available on reasonable request from qualified researchers, in accordance with institutional and ethical regulations. A data sharing plan has been submitted in the trial registry in line with International Committee of Medical Journal Editors requirements.

### Statistical methods

Given the exploratory nature of the trial, analyses will be descriptive. Changes in lesion count, SUV_max_ and SUV_mean_ will be summarised using medians and IQRs. Comparative analyses between the scans performed on day 7 or 14 will be compared with baseline may use the Wilcoxon signed-rank or linear mixed models where appropriate. Results will guide power calculations for subsequent confirmatory trials.

### Data monitoring and auditing

An independent quality-monitoring official will oversee data integrity in accordance with GCP guidelines. Informed consent forms of selected participants will undergo check, and onsite monitoring will entail Source Data Verification to ensure alignment between data recorded in the CRFs and original source documents such as patient records and laboratory results. The intensity of this verification is related to the study’s risks. Monitoring activities will encompass verification of adherence to inclusion and exclusion criteria as well as scrutiny of the primary outcome measure, following a monitoring plan devised by the CRB. Additionally, the monitor will ensure that AEs and SAEs are reported promptly within legislative and regulatory timeframes. Throughout the study duration, the independent monitor will schedule four monitoring visits: the first visit after enrolling 2 patients, the second visit 6 months later, the third visit after 1 year, and finally, the close-out visit.

## Ethics and dissemination

The Medical Ethics Committee (METC) Amsterdam UMC has provided approval for the study protocol (2025–5 20 482-52-03), ensuring compliance with the principles outlined in the Declaration of Helsinki. The participant information sheet and informed consent form are provided as online supplemental material. Any protocol amendments during the study will be subjected to METC review and will be appropriately updated in the relevant registries. Written informed consent will be obtained from each patient prior to their participation. Findings from this study will be submitted for publication in a peer-reviewed journal, regardless of the outcome of this study, adhering to personal data privacy regulations, other applicable legislations, and the Central Committee on Research Involving Human Subjects (Centrale Commissie Mensgebonden Onderzoek). Furthermore, the study results will be disseminated at national and international conferences. All collected data will be archived in an open access digital repository in anonymous form.

## Discussion

The DARO-flare study marks a novel approach to using the darolutamide-induced flare phenomenon as a means to advance molecular imaging and staging methodologies in PCa. To our knowledge, this is the first prospective clinical study specifically designed to investigate and harness this biological effect for diagnostic purposes. Given the scarcity of prior research exploring the clinical translation of PSMA upregulation following short-term AR inhibition, a degree of uncertainty remains regarding both the magnitude and consistency of the flare phenomenon in humans. Consequently, we adopted a pilot, proof-of-concept study design that enables continuous evaluation of feasibility, safety and imaging response.

Previous studies investigating ARTAs, such as enzalutamide and apalutamide, have provided a biological foundation for this study. Both preclinical and clinical data have demonstrated that short-term ARTA administration can lead to upregulation of PSMA expression in PCa.^1^,^17^ The resulting transient increase in PSMA radiotracer uptake, described as the flare phenomenon, appears to occur within 9–14 days. However, evidence supporting this effect has been largely derived from small retrospective series and preclinical studies, and its translational potential for clinical imaging optimisation has not been established.^8^

Moreover, in contrast to enzalutamide and apalutamide, darolutamide exhibits a more unique pharmacokinetic profile, including lower penetration of the blood–brain barrier, minimal induction of major cytochrome P450 systems and limited potential for drug–drug interactions.^22 23^ Despite these distinctions, darolutamide acts through the same fundamental mechanism of AR antagonism and is therefore expected to elicit a similar PSMA-flare response.^22^ However, darolutamide has been reported to exhibit higher affinity for the AR and a longer dissociation half-life compared with enzalutamide and apalutamide, potentially contributing to similar or better upregulation of PSMA.^23^ Its favourable tolerability profile and restricted central nervous system exposure make darolutamide a suitable agent for short-term interventional studies of this nature, by minimising the likelihood of systemic side effects.

Another critical consideration is the biological and clinical interpretation of changes in PSMA uptake. Although increased SUV_max_ on PSMA PET/CT are widely assumed to reflect enhanced PSMA receptor expression, empirical evidence confirming this direct correlation remains limited. Previous studies have furthermore demonstrated that higher SUV_max_ values may correlate with clinically significant and more aggressive PCa.^24^ However, PSMA PET/CT may also show reduced sensitivity in specific histological subtypes, particularly ductal adenocarcinoma, which can be associated with false-negative findings due to low or heterogeneous PSMA expression.^25^ Therefore, both tumour biology and histological subtype may influence baseline uptake and the magnitude of any observed darolutamide-induced changes. As a result, SUV_max_ variations during the flare-phenomenon should be interpreted with caution, as they may reflect a combination of true receptor modulation and underlying characteristics. Additional translational work, including immunohistochemical validation and molecular imaging-pathology correlation, will be necessary to confirm that observed imaging changes correspond to actual modulation of PSMA protein expression and receptor density.

If the DARO-flare phenomenon can be verified and consistently reproduced, it may have several important clinical implications. Short-term pharmacological induction of PSMA expression could improve the sensitivity of PSMA PET/CT imaging, enabling more accurate detection of micro-metastatic disease and refinement of PCa staging. This enhanced sensitivity could influence therapeutic decision-making, particularly in patients with biochemical recurrence or oligometastatic recurrence, where precise delineation of disease burden determines eligibility for metastasis-directed therapy or systemic treatment initiation.

It is equally important to emphasise the exploratory nature of the present study. As a single-centre, phase I–II proof-of-concept study with a limited sample size, the DARO-flare study is not powered to assess clinical outcomes or long-term efficacy. Instead, it aims to establish biological feasibility, characterise the kinetics of PSMA upregulation and inform the design of future multicentre studies. The incorporation of randomisation between days 7 and 14 darolutamide exposure will provide valuable insight into the temporal dynamics of the flare response, allowing subsequent research to define the optimal imaging window.

## Supplementary material

10.1136/bmjopen-2025-115888 online supplemental file 1
