# Supplementary material for: The DARO-flare trial: evaluating the impact of darolutamide on prostate-specific membrane antigen (PSMA) flare and its implications for imaging and staging of hormone-sensitive prostate cancer – study protocol
Source: BMJ Open. 2026 Jun 18;16(6):e115888. doi: 10.1136/bmjopen-2025-115888 (PMC13289025; doi:10.1136/bmjopen-2025-115888)
Supplement: online supplemental file 1 [file bmjopen-16-6-s001.pdf]

Proefpersoneninformatie voor deelname aan medisch-wetenschappelijk onderzoek

DARO-FLARE studie:

Het verhogen van het PSMA-eiwit door darolutamide voor verbetering van de diagnosestelling.

Officiële titel: Klinische toepassing van de androgeenreceptorremmer darolutamide voor het upreguleren van prostaat-specifiek membraanantigeen (PSMA)-eiwitexpressie bij patiënten met hormoongevoelige prostaatkanker ***- de DARO-FLARE-studie -***

Inleiding

Geachte heer,

Met deze informatiebrief willen we u vragen of u wilt meedoen aan medisch-wetenschappelijk onderzoek. Meedoen is vrijwillig. U krijgt deze brief omdat u prostaatkanker heeft.

U leest hier om wat voor onderzoek het gaat, wat het voor u betekent, en wat de voordelen en nadelen zijn. Het is veel informatie. Wilt u de informatie doorlezen en beslissen of u wilt meedoen? Als u wilt meedoen, kunt u het formulier invullen dat u vindt in ***bijlage D***.

Stel uw vragen

U kunt uw beslissing nemen met de informatie die u in deze informatiebrief vindt. Daarnaast raden we u aan om dit te doen:

- Stel vragen aan de onderzoeker die u deze informatie geeft.
- Praat met uw partner, familie of vrienden over dit onderzoek.
- Lees de informatie op www.rijksoverheid.nl/mensenonderzoek.

1. Algemene informatie

Amsterdam UMC, locatie VUmc heeft dit onderzoek opgezet. Hieronder noemen we Amsterdam UMC, locatie VUmc steeds de ‘opdrachtgever’. De studie wordt betaald door het farmaceutisch bedrijf [redacted] Onderzoekers, dit kunnen artsen, arts-onderzoekers of onderzoeksverpleegkundigen zijn, voeren het onderzoek uit. Voor dit onderzoek zijn in totaal 32 deelnemers nodig uit het Amsterdam UMC, welke zijn verdeeld over 4 groepen. De medisch-ethische toetsingscommissie van het Amsterdam UMC heeft dit onderzoek goedgekeurd.

2. Wat is het doel van het onderzoek?

Het doel van het onderzoek is om te achterhalen of het medicijn darolutamide kan zorgen voor een betere opsporing van prostaatkanker. We willen begrijpen hoe darolutamide werkt en of het de diagnose van prostaatkanker kan verbeteren.

3. Wat is de achtergrond van het onderzoek?

Wij benaderen u omdat uw behandelend arts heeft vastgesteld dat u prostaatkanker heeft.

Natuurlijke hormonen spelen een rol bij prostaatkanker. Dit onderzoek bekijkt een medicijn genaamd darolutamide, dat de werking van deze hormonen beïnvloedt. Het medicijn kan een eiwit genaamd PSMA verhogen, wat belangrijk is voor het opsporen van prostaatkanker. Dit kan helpen bij het beter zien van tumoren op speciale beelden (PET-scans).

We denken dat het medicijn, wanneer het kort wordt gebruikt, een verbetering kan geven in het zien van tumoren en het bepalen van de ernst van de ziekte. Dit kan uiteindelijk leiden tot een betere behandeling op maat en een hogere levenskwaliteit voor mensen met prostaatkanker.

4. Hoe verloopt het onderzoek?

Hoelang duurt het onderzoek?
Doet u mee met het onderzoek? Dan duurt dat in totaal ongeveer 3 weken.

Stap 1: bent u geschikt om mee te doen?
We willen eerst weten of u geschikt bent om mee te doen. Daarom doet de onderzoekers een aantal onderzoeken:

- De onderzoeker vraagt naar uw medische geschiedenis.
- De onderzoeker kijkt of het standaard uitgevoerde bloedonderzoek goed is.
- De onderzoeker controleert of eventuele medicijnen die u gebruikt veilig gebruikt kunnen worden tijdens het onderzoek.
- PSMA-PET scan. Deze wordt gemaakt als standaard zorg, als dit nog niet gebeurd is. Deze scan worden gemaakt om de prostaatkanker in beeld te brengen en te bepalen of u het PSMA-eiwit heeft. We zullen hieronder uitleggen hoe een PSMA-PET scan verloopt.

Het kan voorkomen dat u niet geschikt wordt bevonden voor dit onderzoek, dan kunt u niet meedoen.

PSMA PET-scan verloop

- **Voorbereiding**: Een ervaren arts zal bij u een plastic slangetje (infuus) aanleggen in uw arm voor het inspuiten van de radioactief gemerkte stof, welke nodig is voor de scan.

Hierna wordt de radioactief gemerkte stof ingespoten via het infuus en moet u wachten terwijl deze stof zich verspreidt door het lichaam. Dit duurt ongeveer 2 uur.

- **De scan zelf**: Het onderzoek vindt plaats in de PET-CT scanner. De scanner heeft de vorm van een grote staande ring en is ongeveer 50 cm diep. Hij is aan beide kanten open. U zal op een speciale tafel moeten liggen, waarna u vanzelf door de scanner geschoven wordt. Deze maakt nu foto's van de binnenkant van uw lichaam. Het is belangrijk om zo stil mogelijk te blijven liggen.
- **Afloop**: Na ongeveer 30 minuten is de scan klaar en helpt een medewerker u van de tafel. De gemaakte beelden worden door een arts beoordeeld en u kunt weer naar huis.

Stap 2: het gebruik van darolutamide

Indien u geschikt bent voor het onderzoek, zullen de PET-scans worden gepland en het medicijn darolutamide aan u worden gegeven. Darolutamide is een bestaand en goedgekeurd medicijn die wordt gebruikt bij de behandeling van prostaatkanker. In dit onderzoek gebruiken we hetzelfde medicijn, maar voor een ander doel.

In totaal worden 2 verschillende patiëntengroepen met prostaatkanker onderzocht.

- De eerste groep zijn patiënten bij wie op de PET-scan is gezien dat de uitgezaaide kanker zich beperkt tot een klein aantal plekken buiten de prostaat. De groep is al eerder behandeld door een operatie of bestraling.
- De tweede groep zijn patiënten met prostaatkanker bij wie na een operatie om de prostaat te verwijderen, een lichte stijging van het PSA-gehalte in het bloed is gemeten.

Voor dit onderzoek maken we naast de 2 patiëntengroepen, ook 2 behandelgroepen:

- Groep 1. De mensen in deze groep krijgen darolutamide voor 14 dagen.
- Groep 2. De mensen in deze groep krijgen darolutamide voor 7 dagen.

Loting bepaalt in welke groep u komt en hoe lang u darolutamide krijgt.

Stap 3: onderzoeken en metingen

Voor het onderzoek is het nodig dat u 3 keer in 3 weken naar het ziekenhuis komt. De eerste twee bezoeken duren ongeveer 3 uur. Het laatste bezoek duurt ongeveer 30 minuten. We doen de volgende onderzoeken:

- PSMA-PET scan (bij bezoek 1 en 2)
- Checken of u bijwerkingen heeft van darolutamide (bij alle bezoeken)
- U vult een vragenlijst in (alleen bij het laatste bezoek)

In ***bijlage C – Schema met onderzoekshandelingen*** vindt u een overzicht met alle handelingen in het kader van dit onderzoek en voor elke specifieke groep met een bepaalde dosering darolutamide.

Wat is er anders dan bij gewone zorg?

De eerste PSMA-PET scan, voorafgaand aan het onderzoek valt onder de standaardzorg. In dit onderzoek krijgt u een aantal extra onderzoeken en behandelingen die niet bij de standaardzorg horen.

- 2 extra PSMA-PET scans.
- Het medicijn darolutamide

De controles die bij dit onderzoek horen zijn extra. Na het afronden van het onderzoek ondergaat u de standaardzorg.

5. Welke afspraken maken we met u?

We willen graag dat het onderzoek goed verloopt. Daarom maken we de volgende afspraken met u:

- U neemt het medicijn darolutamide op de manier die de onderzoeker u heeft uitgelegd.
- U doet tijdens dit onderzoek niet mee aan een ander medisch-wetenschappelijk onderzoek.
- U komt naar iedere afspraak.
- U draagt de deelnemerskaart van het onderzoek bij u. Bijvoorbeeld in uw portemonnee. Hierop staat dat u meedoet aan dit onderzoek. En wie men moet waarschuwen bij een noodsituatie. Laat deze kaart zien als u bij een andere arts komt.
- U neemt contact op met de onderzoeker in deze situaties:
  - U wilt andere medicijnen gaan gebruiken. Ook als dit homeopathische middelen, natuurgeneesmiddelen, vitaminen of geneesmiddelen van de drogist zijn.
  - U wordt in een ziekenhuis opgenomen of behandeld.
  - U krijgt plotseling problemen met uw gezondheid.
  - U wilt niet meer meedoen met het onderzoek.
  - Uw telefoonnummer, adres of e-mailadres verandert.

Mag uw partner zwanger worden tijdens het onderzoek?
Bent u een man, en heeft u een vrouwelijke partner? Dan moet u ervoor zorgen dat zij niet zwanger kan worden van u. Dit onderzoek kan namelijk gevolgen hebben voor een ongeboren kind. Het is niet bekend welke gevolgen. De onderzoeker vertelt u hoe u het beste een zwangerschap voorkomt. Praat hierover met uw partner.

Toch zwanger?
Wordt uw partner zwanger van u tijdens het onderzoek? Vraag haar dan toestemming om dit aan de onderzoeker te laten weten. Dan kan de zwangerschap extra gecontroleerd worden en kan informatie over het verloop en de uitkomst van de zwangerschap bij andere hulpverleners worden opgevraagd. Maar alleen als u/ uw zwangere partner daar toestemming voor geeft.

6. Van welke bijwerkingen, nadelige effecten of ongemakken kunt u last krijgen?

Bijwerkingen van medicijnen

Het onderzoeksmiddel darolutamide kan bijwerkingen geven. Bij kortdurend gebruik van darolutamide worden alleen milde bijwerkingen verwacht. Hieronder vallen vermoeidheid, spijsverteringsproblemen zoals misselijkheid of diarree, zwakte en spierpijn. Het is belangrijk om te weten dat niet iedereen deze bijwerkingen zal ervaren en dat ze meestal weer verdwijnen als u stopt met het medicijn. Meer informatie over darolutamide staat in de bijsluiter, zie ***bijlage E***.

De radioactief gemerkte stof die we toedienen tijdens de PET-scan kan ook bijwerkingen hebben, hoewel deze bijna niet voorkomen. De bijwerkingen kunnen zijn: vermoeidheid, verandering in smaak en hoofdpijn. Deze bijwerkingen komen voor bij minder dan één op de tien mensen. Uw gezondheid wordt nauwlettend in de gaten gehouden en als u zich ooit zorgen maakt over bijwerkingen, kunt u dit altijd met uw arts bespreken. Uw welzijn staat voorop.

Wat zijn de nadelen van onderzoeken die gebruik maken van straling?
Tijdens dit onderzoek maken we gebruik van röntgenstraling en radioactieve stoffen. In dit onderzoek krijgt u in totaal ongeveer 19 mSv aan straling voor de 2 PSMA-PET scans. Ter vergelijking: de ‘gewone’ straling die iedereen in Nederland krijgt, is ongeveer 2,9 mSv per jaar. Het kan geen kwaad als u voor een medische reden een onderzoek of behandeling met straling moet ondergaan.

- Krijgt u vaker een onderzoek met straling? Bespreek dan met de onderzoeker of het verstandig is dat u meedoet.
- De straling die we tijdens het onderzoek gebruiken kan op latere leeftijd leiden tot schade aan uw gezondheid. Maar dit is een klein risico. Wel adviseren we u de komende tijd niet nog een keer mee te doen aan een wetenschappelijk onderzoek met straling.

7. Wat zijn de voordelen en de nadelen als u meedoet aan het onderzoek?

Meedoen aan het onderzoek kan voordelen en nadelen hebben. Hieronder zetten we ze op een rij. Denk hier goed over na, en praat erover met anderen.

Meedoen aan het onderzoek kan deze voordelen hebben:

- Het verhogen van het PSMA-eiwit kan zorgen voor een betere opsporing van de mate prostaatkanker, maar zeker is dat niet. Als er bij het onderzoek meer uitzaaiingen worden gezien, zal dit de behandeling ook kunnen beïnvloeden.
- Met uw deelname helpt u de onderzoekers om meer inzicht te krijgen in het opsporen van prostaatkanker.

Meedoen aan het onderzoek kan deze nadelen of gevolgen hebben:

- U kunt last krijgen van de bijwerkingen of nadelige effecten van darolutamide.
- U kunt last hebben van de extra PSMA-PET scans tijdens het onderzoek.
- U moet zich houden aan de afspraken die horen bij het onderzoek.

- Het is mogelijk dat er tijdens de PET/CT scan toevallig iets wordt ontdekt dat niet direct van belang is voor het onderzoek maar wel voor uw gezondheid of die van uw familieleden. Zie ook paragraaf 10 over onverwachte ontdekkingen.

Wilt u niet meedoen?
U beslist zelf of u meedoet aan het onderzoek. Wilt u niet meedoen? Dan krijgt u de gewone behandeling voor prostaatkanker. Uw arts kan u meer vertellen over de behandelingsmogelijkheden die er zijn. En over de voor- en nadelen daarvan.

8. Wanneer stopt het onderzoek?

De onderzoeker laat het u weten als er nieuwe informatie over het onderzoek komt die belangrijk voor u is. De onderzoeker vraagt u daarna of u blijft meedoen.

In deze situaties stopt voor u het onderzoek:

- Alle onderzoeken volgens het schema zijn voorbij.
- Het einde van het hele onderzoek is bereikt.
- U wilt zelf stoppen met het onderzoek. Dat mag op ieder moment. Meld dit dan meteen bij de onderzoeker. U hoeft er niet bij te vertellen waarom u stopt. De onderzoeker kan voor uw veiligheid nog een of meer controles afspreken.
- De onderzoeker vindt het beter voor u om te stoppen. De onderzoeker zal u nog wel uitnodigen voor een nacontrole.
- Een van de volgende instanties besluit dat het onderzoek moet stoppen:
  - Amsterdam UMC, locatie VUmc
  - de overheid, of
  - de medisch-ethische commissie die het onderzoek beoordeelt.

Wat gebeurt er als u stopt met het onderzoek?
De onderzoekers gebruiken de gegevens die tot het moment van stoppen zijn verzameld. Het hele onderzoek is afgelopen als alle deelnemers klaar zijn.

9. Wat gebeurt er na het onderzoek?

Krijgt u de resultaten van het onderzoek?
Ongeveer 1 jaar nadat het onderzoek is afgerond laat de onderzoeker u weten wat de belangrijkste uitkomsten zijn van het onderzoek. Wilt u dit niet weten? Zeg dat dan tegen de onderzoeker. Hij/zij zal het u dan niet vertellen.

10. Wat doen we met uw gegevens?

Doet u mee met het onderzoek? Dan geeft u ook toestemming om uw gegevens te verzamelen, gebruiken en bewaren.

Welke gegevens bewaren we?
We bewaren deze gegevens:

- uw naam
- uw adres
- uw geboortedatum
- gegevens over uw gezondheid
- (medische) gegevens die we tijdens het onderzoek verzamelen.

Waarom verzamelen, gebruiken en bewaren we uw gegevens?

We verzamelen, gebruiken en bewaren uw gegevens om de vragen van dit onderzoek te kunnen beantwoorden. En om de resultaten te kunnen publiceren.

Hoe beschermen we uw privacy?

Om uw privacy te beschermen geven wij uw gegevens een code. Op al uw gegevens zetten we alleen deze code. De sleutel van de code bewaren we op een beveiligde plek in het ziekenhuis. Als we uw gegevens verwerken, gebruiken we steeds alleen die code. Ook in rapporten en publicaties over het onderzoek kan niemand terughalen dat het over u ging.

Wie kunnen uw gegevens zien?

Sommige personen kunnen wel uw naam en andere persoonlijke gegevens zonder code inzien. Dit kunnen gegevens zijn die speciaal voor dit onderzoek zijn verzameld, maar ook gegevens uit uw medisch dossier.
Dit zijn mensen die controleren of de onderzoekers het onderzoek goed en betrouwbaar uitvoeren. Deze personen kunnen bij uw gegevens komen:

- Leden van de commissie die de veiligheid van het onderzoek in de gaten houdt.
- Een controleur die door de opdrachtgever is ingehuurd.
- Nationale en internationale toezichthoudende autoriteiten.

Deze personen houden uw gegevens geheim. Voor inzage door deze personen vragen wij u toestemming te geven. De Inspectie Gezondheidszorg en Jeugd kan zonder uw toestemming uw gegevens inzien.

Hoelang bewaren we uw gegevens?

We bewaren uw gegevens 25 jaar in het ziekenhuis.

Mogen we uw gegevens gebruiken voor ander onderzoek?

Uw verzamelde gegevens kunnen ook van belang zijn voor ander wetenschappelijk onderzoek op het gebied van prostaatkanker en/of de behandelmethode. Daarvoor zullen uw gegevens 25 jaar worden bewaard in het ziekenhuis. In het toestemmingformulier geeft u aan of u dit goed vindt. Geeft u geen toestemming? Dan kunt u nog steeds meedoen met dit onderzoek. U krijgt dezelfde zorg.

Wat gebeurt er bij onverwachte ontdekkingen?
Tijdens het onderzoek kunnen we toevallig iets vinden dat niet direct van belang is voor het onderzoek maar wel voor uw gezondheid of voor de gezondheid van uw familieleden. De onderzoeker neemt dat contact op met uw behandelend arts. U bespreekt dan met uw specialist wat er moet gebeuren. De kosten hiervan vallen onder uw eigen zorgverzekering. U geeft met het formulier toestemming voor het informeren van uw specialist.

Kunt u uw toestemming voor het gebruik van uw gegevens weer intrekken?
U kunt uw toestemming voor het gebruik van uw gegevens op ieder moment intrekken. Zeg dat dan tegen de onderzoeker. Dit geldt voor het gebruik in dit onderzoek en voor het gebruik in ander onderzoek. Maar let op: trekt u uw toestemming in, en hebben onderzoekers dan al gegevens verzameld voor een onderzoek? Dan mogen zij deze gegevens nog wel gebruiken.

Wilt u meer weten over uw privacy?

- Wilt u meer weten over uw rechten bij de verwerking van persoonsgegevens? Kijk dan op www.autoriteitpersoonsgegevens.nl.
- Heeft u vragen over uw rechten? Of heeft u een klacht over de verwerking van uw persoonsgegevens? Neem dan contact op met degene die verantwoordelijk is voor de verwerking van uw persoonsgegevens. Voor uw onderzoek is dat:
    - Amsterdam UMC. Zie bijlage A voor contactgegevens, en website.
- Als u klachten heeft over de verwerking van uw persoonsgegevens, raden we u aan om deze eerst te bespreken met het onderzoeksteam. U kunt ook naar de Functionaris Gegevensbescherming van Amsterdam UMC gaan. Of u dient een klacht in bij de Autoriteit Persoonsgegevens.

Waar vindt u meer informatie over het onderzoek?
Op de volgende website(s) vindt u meer informatie over het onderzoek.
www.onderzoekmetmensen.nl, https://euclinicaltrials.eu. Na het onderzoek kan de website een samenvatting van de resultaten van dit onderzoek tonen. U vindt het onderzoek door te zoeken op nummer: 2025-520482-52-02.

11. Krijgt u een vergoeding als u meedoet aan het onderzoek?

De onderzoeksmiddelen (darolutamide en de radioactief gemerkte stof) en de extra PSMA-PET scans kosten u niets. U krijgt ook geen vergoeding als u meedoet aan dit onderzoek. Wel krijgt u vergoeding voor uw (extra) reiskosten.

12. Bent u verzekerd tijdens het onderzoek?

Voor iedereen die meedoet aan dit onderzoek is een verzekering afgesloten. De verzekering betaalt voor schade door het onderzoek. Maar niet voor alle schade. In ***bijlage B*** vindt u meer informatie over de verzekering en de uitzonderingen. Daar staat ook aan wie u schade kunt melden.

13. We informeren uw huisarts en/of behandelend specialist en/of apotheker

De onderzoeker stuur uw huisarts een brief om te laten weten dat u meedoet aan het onderzoek. Dit is voor uw eigen veiligheid.

14. Heeft u vragen?

Vragen over het onderzoek kunt u steller aan de onderzoeker, Dr. André N. Vis, uroloog (tel via secretariaat urologie [redacted]

Heeft u een klacht? Bespreek dit dan met de onderzoeker of de arts die u behandelt. Wilt u dit liever niet? Ga dan naar het Servicecentrum patiënt & zorgverlener van het VUmc Alle gegevens vindt u in **bijlage A**: Contactgegevens

15. Hoe geeft u toestemming voor het onderzoek?

U kunt eerst rustig nadenken over dit onderzoek. De minimale bedenktijd om wel of niet deel te nemen aan de studie is 48 uur. Daarna vertelt u de onderzoeker of u de informatie begrijpt en of u wel of niet wilt meedoen. Wilt u meedoen? Dan vult u het toestemmingsformulier in dat u bij deze informatiebrief vindt. U en de onderzoeker krijgen allebei een getekende versie van deze toestemmingsverklaring.

Dank voor uw tijd.

16. Bijlagen

A. Contactgegevens

B. Informatie over de verzekering

C. Schema met onderzoekshandelingen

D. Toestemmingsformulier

E. Bijsluiter darolutamide

Bijlage A: Contactpersonen

Onderzoekers Amsterdam UMC, locatie VUmc:

Dr. AN Vis, *uroloog*
t.a.v. secretariaat urologie kantooruren tel: [redacted]
De Boelelaan 1117
Postbus 7057, 1007 MB Amsterdam

Drs. Suzanne van der Gaag, *uitvoerend onderzoeker*
secretariaat nucleaire geneeskunde kantooruren tel: [redacted]
De Boelelaan 1117
Postbus 7057, 1007 MB Amsterdam

Klachten:
Patiëntenservice Zorgsupport locatie VUmc bereikbaar via [redacted]

Functionaris voor de Gegevensbescherming van het Amsterdam UMC bereikbaar via
[redacted]

Bijlage B: informatie over de verzekering

De opdrachtgever Amsterdam UMC heeft een verzekering afgesloten voor iedereen die meedoet aan het onderzoek. De verzekering betaalt de schade die u heeft doordat u aan het onderzoek meedeed. Het gaat om schade die u krijgt tijdens het onderzoek, of binnen 4 jaar na het einde van uw deelname aan het onderzoek. U moet schade binnen deze 4 jaar melden bij de verzekeraar.

Heeft u schade door het onderzoek? Meld dit dan bij deze verzekeraar:

De verzekeraar van het onderzoek is:	
Naam verzekeraar:	Centramed B.A.
Adres:	Postbus 7374<br>2701 AJ Zoetermeer
Telefoonnummer:	070 301 70 70
E-mail:	info@centramed.nl
Polisnummer:	[redacted]

[redacted]

Let op: de verzekering dekt de volgende schade **niet**:

- Schade door een risico waarover we u informatie hebben gegeven in deze brief. Maar dit geldt niet als het risico groter bleek te zijn dan we van tevoren dachten. Of als het risico heel onwaarschijnlijk was.
- Schade aan uw gezondheid die ook zou zijn ontstaan als u niet aan het onderzoek had meegedaan.
- Schade die ontstaat doordat u aanwijzingen of instructies niet of niet goed opvolgde.

Deze bepalingen staan in het 'Besluit verplichte verzekering bij medisch-wetenschappelijk onderzoek met mensen 2015'. Dit besluit staat in de Wettenbank van de overheid (https://wetten.overheid.nl).

Bijlage C: schema met onderzoekshandelingen

	Startdag	Dag 7	Dag 14	Dag 21
**Check voorwaarden deelname**	✓			
**Medische geschiedenis** *(uit dossier)*	✓ #			
**Bloedonderzoek** *(uit dossier)*	✓ #			
**PSMA PET-scan**	✓ #	✓	✓	
**Kwaliteit van leven vragenlijst**	✓			✓
**Start medicijn darolutamide**	✓			
**Bijwerkingen check**		✓	✓	✓

Dit valt onder standaardzorg.

Toestemmingsformulier proefpersoon

De DARO-FLARE studie

Het verhogen van het PSMA-eiwit door darolutamide voor verbetering van de diagnosestelling

- Ik heb de informatiebrief gelezen. Ook kon ik vragen stellen. Mijn vragen zijn goed genoeg beantwoord. Ik had genoeg tijd om te beslissen of ik meedoe.
- Ik weet dat meedoen vrijwillig is. Ook weet ik dat ik op ieder moment kan beslissen om toch niet mee te doen met het onderzoek. Of om ermee te stoppen. Ik hoef dan niet te zeggen waarom ik wil stoppen.
- Ik geef de onderzoeker toestemming om huisarts en apotheker te laten weten dat ik meedoe aan dit onderzoek.
- Ik geef de onderzoeker toestemming om mijn specialist informatie te geven over onverwachte bevindingen uit het onderzoek die van belang zijn voor mijn gezondheid.
- Ik geef de onderzoekers toestemming om mijn gegevens te verzamelen en gebruiken. De onderzoekers doen dit alleen om de onderzoeksvraag van dit onderzoek te beantwoorden.
- Ik weet dat voor de controle van het onderzoek sommige mensen al mijn gegevens kunnen inzien. Die mensen staan in deze informatiebrief. Ik geef deze mensen toestemming om mijn gegevens in te zien voor deze controle.
- Ik weet dat ik mijn partner niet zwanger mag maken tijdens het onderzoek.
- De onderzoeker heeft met mij besproken hoe ik het beste voorkom dat mijn partner zwanger wordt.

- Wilt u in de tabel hieronder ja of nee aankruisen?

Ik geef toestemming om mijn gegevens te bewaren om dit te gebruiken voor ander onderzoek, zoals in de informatiebrief staat.	Ja ☐	Nee☐
Ik geef toestemming om mij eventueel na dit onderzoek te vragen of ik wil meedoen met een vervolgonderzoek.	Ja ☐	Nee☐

- Ik wil meedoen aan dit onderzoek.

Mijn naam is (deelnemer): ………………………………..
Handtekening: ………………………   Datum : __ / __ / __

Ik verklaar dat ik deze deelnemer volledig heb geïnformeerd over het genoemde onderzoek.

Wordt er tijdens het onderzoek informatie bekend die die de toestemming van de deelnemer kan beïnvloeden? Dan laat ik dit op tijd weten aan deze deelnemer.

Naam onderzoeker (of diens vertegenwoordiger):……………………………….
Handtekening:………………………   Datum: __ / __ / __

De deelnemer krijgt een volledige informatiebrief mee, samen met een getekende versie van het toestemmingsformulier.

Bijlage E: Bijsluiter Darolutamide

Bijsluiter: informatie voor de patiënt

NUBEQA 300 mg filmomhulde tabletten
darolutamide

Lees goed de hele bijsluiter voordat u dit geneesmiddel gaat innemen want er staat belangrijke informatie in voor u.

- Bewaar deze bijsluiter. Misschien heeft u hem later weer nodig.
- Heeft u nog vragen? Neem dan contact op met uw arts of apotheker.
- Geef dit geneesmiddel niet door aan anderen, want het is alleen aan u voorgeschreven. Het kan schadelijk zijn voor anderen, ook al hebben zij dezelfde klachten als u.
- Krijgt u last van de bijwerkingen die in rubriek 4 staan? Of krijgt u een bijwerking die niet in deze bijsluiter staat? Neem dan contact op met uw arts of apotheker.

Inhoud van deze bijsluiter

1. Wat is NUBEQA en waarvoor wordt dit middel ingenomen?
2. Wanneer mag u dit middel niet innemen of moet u er extra voorzichtig mee zijn?
3. Hoe neemt u dit middel in?
4. Mogelijke bijwerkingen
5. Hoe bewaart u dit middel?
6. Inhoud van de verpakking en overige informatie

1. Wat is NUBEQA en waarvoor wordt dit middel ingenomen?

NUBEQA bevat de werkzame stof darolutamide.
Het wordt gebruikt voor de behandeling van **volwassen mannen met prostaatkanker**:

- bij wie de prostaatkanker zich niet naar andere delen van het lichaam heeft verspreid en bij wie de prostaatkanker niet meer reageert op medicamenteuze of chirurgische behandeling die het testosterongehalte verlaagt (dit wordt ook niet-gemetastaseerde castratieresistente prostaatkanker genoemd)

of

- bij wie de prostaatkanker zich wel naar andere delen van het lichaam heeft verspreid en reageert op medische of chirurgische behandeling die het testosterongehalte omlaag brengt (dit wordt ook gemetastaseerde hormoongevoelige prostaatkanker genoemd).
  Hiervoor kan uw arts u ook docetaxel geven.

Hoe werkt NUBEQA?
NUBEQA blokkeert de activiteit van de mannelijke geslachtshormonen, genaamd androgenen, zoals testosteron. Androgenen kunnen ervoor zorgen dat de prostaatkanker groeit. Door het blokkeren van deze hormonen zorgt darolutamide ervoor dat prostaatkankercellen niet meer groeien en delen.

2. Wanneer mag u dit middel niet innemen of moet u er extra voorzichtig mee zijn?

Wanneer mag u dit middel niet gebruiken?

- U bent allergisch voor een van de stoffen in dit geneesmiddel. Deze stoffen kunt u vinden in rubriek 6.
- U bent een vrouw die zwanger is of zwanger kan worden.

Wanneer moet u extra voorzichtig zijn met dit middel?
Neem contact op met uw arts of apotheker voordat u dit middel inneemt als:
- u problemen heeft met uw nieren
- u problemen heeft met uw lever
- u hartaandoeningen heeft, waaronder problemen met het hartritme, of als u geneesmiddelen voor deze aandoeningen gebruikt
- u een operatie heeft gehad voor aandoeningen aan uw bloedvaten.

Het gebruik van dit middel kan uw leverfunctie beïnvloeden. Als uw bloedonderzoek abnormale resultaten van uw leverfunctie laat zien, kan uw arts besluiten dat u definitief moet stoppen met het innemen van dit middel.

Kinderen en jongeren tot 18 jaar
Dit geneesmiddel is niet voor gebruik bij kinderen en jongeren tot 18 jaar. Prostaatkanker komt in deze leeftijdsgroep niet voor.

Gebruikt u nog andere geneesmiddelen?
Neemt u naast NUBEQA nog andere geneesmiddelen in, heeft u dat kort geleden gedaan of bestaat de mogelijkheid dat u binnenkort andere geneesmiddelen gaat innemen? Vertel dat dan uw arts of apotheker.

De volgende geneesmiddelen kunnen de werking van NUBEQA beïnvloeden of NUBEQA kan de werking beïnvloeden van deze geneesmiddelen. Deze geneesmiddelen worden gewoonlijk gebruikt voor de behandeling van:
- bacteriële infecties, zoals **rifampicine**
- epilepsie, zoals **carbamazepine**, **fenobarbital**, **fenytoïne**
- een licht neerslachtige stemming en milde angst: **sint-janskruid** (een kruidengeneesmiddel)
- een hoog cholesterol, zoals **rosuvastatine**, **fluvastatine**, **atorvastatine**, **pitavastatine**
- ernstige gewrichtsontsteking, ernstige gevallen van de huidziekte psoriasis en kanker: **methotrexaat**
- inflammatoire darmziekten: **sulfasalazine**

Uw arts kan om die reden de dosis aanpassen van de geneesmiddelen die u gebruikt.

Zwangerschap, borstvoeding en vruchtbaarheid
NUBEQA is niet bestemd voor gebruik bij vrouwen.
Dit geneesmiddel zou de mannelijke vruchtbaarheid kunnen verminderen.
Volg tijdens uw behandeling en gedurende 1 week na stopzetting van de behandeling de volgende adviezen op:
- gebruik een condoom of een ander zeer effectief voorbehoedsmiddel om zwangerschap te voorkomen als u geslachtsgemeenschap heeft met een vrouw die zwanger kan worden. Vraag uw arts wat het beste voorbehoedsmiddel voor u is.
- gebruik een condoom om het ongeboren kind te beschermen als u geslachtsgemeenschap heeft met een zwangere vrouw.

Rijvaardigheid en het gebruik van machines
Het is onwaarschijnlijk dat dit geneesmiddel een negatieve invloed heeft op uw rijvaardigheid of uw vermogen om machines te bedienen.

NUBEQA bevat lactose
Indien uw arts u heeft meegedeeld dat u bepaalde suikers niet verdraagt, neem dan contact op met uw arts voordat u dit middel inneemt.

3. Hoe neemt u dit middel in?

Neem dit geneesmiddel altijd in precies zoals uw arts of apotheker u dat heeft verteld. Twijfelt u over het juiste gebruik? Neem dan contact op met uw arts of apotheker.

De aanbevolen dosering is
Tweemaal daags 2 tabletten. Neem niet meer dan 4 tabletten per dag in.

Uw arts kan uw dosis verlagen tot tweemaal daags 1 tablet als u problemen met uw lever of nieren heeft.

Innemen van NUBEQA
Slik de tabletten in hun geheel door – u mag ze niet breken of vermalen. Neem de tabletten in met voedsel en een glas water.

Uw arts kan u ook andere geneesmiddelen voorschrijven terwijl u NUBEQA inneemt.

Heeft u te veel van dit middel ingenomen?
Ga door met de behandeling en neem de volgende dosis zoals gepland.

Bent u vergeten dit middel in te nemen?
Neem uw overgeslagen dosis in zodra u eraan denkt vóór de volgende geplande dosis. Neem geen dubbele dosis om één of meer vergeten tabletten in te halen.

Als u stopt met het innemen van dit middel
Stop niet met het innemen van dit geneesmiddel, tenzij uw arts u dit heeft verteld.

Heeft u nog andere vragen over het gebruik van dit geneesmiddel? Neem dan contact op met uw arts of apotheker.

4. Mogelijke bijwerkingen

Zoals elk geneesmiddel kan ook dit geneesmiddel bijwerkingen hebben, al krijgt niet iedereen daarmee te maken.

Bijwerkingen van NUBEQA komen voor met de volgende frequenties:
Zeer vaak voorkomende bijwerkingen (kunnen voorkomen bij meer dan 1 op de 10 gebruikers):
- vermoeidheid
- een verlaagd aantal van een bepaald soort witte bloedcellen die neutrofielen worden genoemd. Dit blijkt uit bloedonderzoek
- hogere spiegels van stoffen die door de lever worden aangemaakt: bilirubine, alanine-transaminase en aspartaat-transaminase. Dit blijkt uit bloedonderzoek.

Vaak voorkomende bijwerkingen (kunnen voorkomen bij minder dan 1 op de 10 gebruikers):
- blokkade van de slagaders in het hart
- hartfalen
- huiduitslag
- pijn in armen en benen
- botbreuken

Bij gebruik in combinatie met docetaxel komen bijwerkingen van NUBEQA voor met de volgende frequenties:
Zeer vaak voorkomende bijwerkingen (kunnen voorkomen bij meer dan 1 op de 10 gebruikers):
- hoge bloeddruk
- huiduitslag
- een verlaagd aantal van een bepaald soort witte bloedcellen die neutrofielen worden genoemd. Dit blijkt uit bloedonderzoek
- hogere spiegels van stoffen die door de lever worden aangemaakt: bilirubine, alanine-transaminase en aspartaat-transaminase. Dit blijkt uit bloedonderzoek.

Vaak voorkomende bijwerkingen (kunnen voorkomen bij minder dan 1 op de 10 gebruikers):
- botbreuken
- borstontwikkeling bij mannen

Het melden van bijwerkingen
Krijgt u last van bijwerkingen, neem dan contact op met uw arts of apotheker. Dit geldt ook voor mogelijke bijwerkingen die niet in deze bijsluiter staan. U kunt bijwerkingen ook rechtstreeks melden via het nationale meldsysteem zoals vermeld in aanhangsel V. Door bijwerkingen te melden, kunt u ons helpen meer informatie te verkrijgen over de veiligheid van dit geneesmiddel.

5. Hoe bewaart u dit middel?

Buiten het zicht en bereik van kinderen houden.

Gebruik dit geneesmiddel niet meer na de uiterste houdbaarheidsdatum. Die vindt u op de doos en op elke blisterverpakking na EXP. Daar staat een maand en een jaar. De laatste dag van die maand is de uiterste houdbaarheidsdatum.

Voor dit geneesmiddel zijn er geen speciale bewaarcondities.

Spoel geneesmiddelen niet door de gootsteen of de WC en gooi ze niet in de vuilnisbak. Vraag uw apotheker wat u met geneesmiddelen moet doen die u niet meer gebruikt. Als u geneesmiddelen op de juiste manier afvoert worden ze op een verantwoorde manier vernietigd en komen ze niet in het milieu terecht.

6. Inhoud van de verpakking en overige informatie

Welke stoffen zitten er in dit middel?
De werkzame stof in dit middel is darolutamide. Elke filmomhulde tablet bevat 300 mg darolutamide.
De andere stoffen in dit middel zijn:
- calciumwaterstoffosfaat (E 341)
- croscarmellosenatrium
- hypromellose
- lactosemonohydraat
- macrogol (E 1521)
- magnesiumstearaat (E 470b)
- povidon (E 1201)
- titaniumdioxide (E 171)

Zie “NUBEQA bevat lactose” in rubriek 2 voor meer informatie.

Hoe ziet NUBEQA eruit en hoeveel zit er in een verpakking?
De filmomhulde tabletten (tabletten) zijn witte tot gebroken witte, ovale tabletten met een lengte van 16 mm en een breedte van 8 mm, waarop aan één kant "300" en aan de andere kant "BAYER" is aangebracht.

Elk doosje bevat 112 filmomhulde tabletten en bestaat uit 7 blisterverpakkingen met elk 16 filmomhulde tabletten.

Houder van de vergunning voor het in de handel brengen
Bayer AG
51368 Leverkusen
Duitsland

Fabrikant

De fabrikant kan worden geïdentificeerd door middel van het batch nummer dat op de doos en op elke blisterverpakking is gedrukt:

- Als de tekens alleen uit cijfers bestaan, dan is de fabrikant
  Orion Corporation, Orion Pharma
  24100 Salo
  Finland

- Als de eerste en tweede tekens BX zijn, dan is de fabrikant
  Bayer AG
  Kaiser-Wilhelm-Allee
  51368 Leverkusen
  Duitsland

Neem voor alle informatie over dit geneesmiddel contact op met de lokale vertegenwoordiger van de houder van de vergunning voor het in de handel brengen:

België/Belgique/Belgien
Bayer SA-NV
Tél/Tel: +32-(0)2-5356311

България
Байер България ЕООД
Тел.: +359 (0)2 424 72 80

Česká republika
Bayer s.r.o.
Tel: +420 266 101 111

Danmark
Bayer A/S
Tlf: +45 45 23 50 00

Deutschland
Bayer Vital GmbH
Tel: +49 (0)214-30 513 48

Eesti
Bayer OÜ
Tel: +372 655 8565

Ελλάδα
Bayer Ελλάς ABEE
Τηλ: +30 210-618 75 00

España
Bayer Hispania S.L.
Tel: +34-93-495 65 00

France
Bayer HealthCare
Tél (N° vert): +33-(0)800 87 54 54

Hrvatska
Bayer d.o.o.
Tel: +385-(0)1-6599 900

Ireland
Bayer Limited
Tel: +353 1 216 3300

Ísland
Icepharma hf.
Sími: +354 540 8000

Italia
Bayer S.p.A.
Tel: +39 02 397 81

Κύπρος
NOVAGEM Limited
Τηλ: +357 22 48 38 58

Latvija
SIA Bayer
Tel: +371 67 84 55 63

Lietuva
UAB Bayer
Tel. +37 05 23 36 868

Luxembourg/Luxemburg
Bayer SA-NV
Tél/Tel: +32(0)2 -535 63 11

Magyarország
Bayer Hungária KFT
Tel:+36 14 87-41 00

Malta
Alfred Gera and Sons Ltd.
Tel: +35 621 44 62 05

Nederland
Bayer B.V.
Tel: +31-23-799 1000

Norge
Bayer AS
Tlf: +47 24 11 18 00

Österreich
Bayer Austria Ges.m.b.H.
Tel: +43-(0)1-711 46-0

Polska
Bayer Sp. z.o.o.
Tel: +48 22 572 35 00

Portugal
Bayer Portugal, Lda.
Tel: +351 21 416 42 00

România
SC Bayer s.r.l.
Tel: +40 21 529 59 00

Slovenija
Bayer d. o. o.
Tel: +386 1 58 14 400

Slovenská republika
Bayer spol. s r.o.
Tel. +421 2 59 21 31 11

Suomi/Finland
Bayer Oy
Puh/Tel: +358 20 785 21

Sverige
Bayer AB
Tel: +46 (0) 8 580 223 00

Deze bijsluiter is voor het laatst goedgekeurd in.

Meer informatie over dit geneesmiddel is beschikbaar op de website van het Europees Geneesmiddelenbureau: https://www.ema.europa.eu.
