# Supplementary material for: The DARO-flare trial: evaluating the impact of darolutamide on prostate-specific membrane antigen (PSMA) flare and its implications for imaging and staging of hormone-sensitive prostate cancer – study protocol
Source: BMJ Open. 2026 Jun 18;16(6):e115888. doi: 10.1136/bmjopen-2025-115888 (PMC13289025; doi:10.1136/bmjopen-2025-115888)
Supplement: Supplementary file 2 [file bmjopen-2025-115888.reviewer_comments.pdf]

PEER REVIEW HISTORY

BMJ Open publishes all reviews undertaken for accepted manuscripts. Reviewers are asked to complete a checklist review form and are provided with free text boxes to elaborate on their assessment. These free text comments are reproduced below.

ARTICLE DETAILS

Title (Provisional)

The DARO-flare Trial: Evaluating the Impact of Darolutamide on Prostate-Specific Membrane Antigen (PSMA) Flare and its Implications for Imaging and Staging of Hormone-Sensitive Prostate Cancer -Study protocol

Authors

van der Gaag, Suzanne; Vis, André; Bartelink, Imke; Hendrikse, Harry; Oprea-Lager, Daniela E.

VERSION 1 - REVIEW

**Reviewer**	**1**
**Name**	**Pepe, Pietro**
**Affiliation**	**Cannizzaro Hospital, Urology**
**Date**	**17-Jan-2026**
**COI**	**None**

The study is interesting but some points could be improved

1. The patients underwent PSMA PET/CT or other clinical staging before definitive treatment? Could add clinical and histological parameters (acinar, ductal, cribiform) of the patients?

2. The Authors should report in the Discussion the SUVmax accuracy in detecting aggressiveness PCa (Pepe P, Pepe L, Tamburo M, Marletta G, Savoca F, Pennisi M, Fraggetta F. 68Ga-PSMA PET/CT and Prostate Cancer Diagnosis: Which SUVmax Value? In Vivo. 2023 May-Jun;37(3):1318-1322. doi: 10.21873/invivo.13211)

3. The Authors should report in the Discussion the false negative rate of PSMA PET/CT in diagnosing and staging ductal prostatic cancer (Pepe P, Pepe L, Curduman M, Pennisi M, Fraggetta F. Ductal prostate cancer staging: Role of PSMA PET/CT. Arch Ital Urol Androl. 2024 Feb 16;96(1):12132. doi: 10.4081/aiua.2024.12132)

**Reviewer**	**2**

**Name**	**Zhu, Xiaofei**
**Affiliation**	**Changhai Hospital, Radiation Oncology**
**Date**	**18-Feb-2026**
**COI**	**None**

The author has submitted two versions of manuscript. One was tracked-changes version and the other was clean version. It seemed that the manuscript has been previously reviewed. And the authors have made substantial revisions. I have one comment. The participants would receive three scans of PSMA-PETCT within two to three weeks. Therefore, the patients would receive excessive radiation exposure. The authors should clarfiy what they would do to reduce the potential risk ascribed to excessive radiation exposure in details.

**Reviewer**	**3**
**Name**	**Singh, Prabhjot**
**Affiliation**	**All India Institute of Medical Sciences, Urology**
**Date**	**09-May-2026**
**COI**	**None**

Well written protocol

VERSION 1 - AUTHOR RESPONSE

Reviewer #1

**Comment**	**Response**
The study is interesting, but some points could be improved:<br>1. The patients underwent PSMA PET/CT or other clinical staging before definitive treatment? Could add clinical and histological parameters (acinar, ductal, cribiform) of the patients?	We thank the reviewer for this suggestion. We have clarified that baseline staging was performed according to standard clinical practice and added documentation of relevant histopathological features.<br><br>*[Line number 118-120]*
2. The authors should report in the discussion the SUVmax accuracy in detecting aggressiveness PCa (Pepe P, Pepe L, Tamburo M, Marletta G, Savoca F, Pennisi M, Fraggetta F. 68Ga-PSMA PET/CT and Prostate Cancer Diagnosis: Which SUVmax Value? In vivo. 2023 May-Jun;37(3):1318-1322. Doi: 10.21873/invivo.13211)	We thank the reviewer for highlighting the relevance of SUV_{max} in relation to tumor aggressiveness. We have incorporated this into the discussion together with its implications for interpretation of PSMA PET changes.<br><br>*[Line number 292-299]*
3. The authors should report in the discussion the false negative rate of PSMA PET/CT in diagnosing and staging ductal prostatic cancer (Pepe P, Pepe L, Curduman M, Pennisi M, Fraggetta F. Ductal prostate cancer staging: Role of PSMA PET/CT.	We thank the reviewer for this important point. We have added a limitation regarding reduced sensitivity of PSMA PET/CT in ductal prostate cancer and its relevance of interpretation of imaging findings.<br><br>*[Line number 292-299]*

Arch Ital Urol Androl. 2024 Feb 16;96(1):12132. Doi: 10.4081/aiua.2024/12132)	

Reviewer #2

Comment	Response
The participants would receive three scans of PSMA PET/CT within two of three weeks. Therefore, the patients would receive excessive radiation exposure. The authors should clarify what they would do to reduce the potential risk ascribed to excessive radiation exposure in details.	We thank the reviewer for this important safety consideration. Radiation exposure is minimized by using low-dose CT protocols and optimized PET acquisition in accordance with ALARA principles. The cumulative radiation exposure associated with the three scans was reviewed and approved by the Medical Ethics Committee and considered acceptable for this short-term study.<br><br>*[Line number 190-192]*

Reviewer #3

Comment	Response
Well written protocol.	We sincerely thank the reviewer for this positive assessment of our manuscript.
